# Antimicrobial Resistance and Molecular Epidemiology of Uropathogenic *Escherichia coli* Isolated From Female Patients in Shanghai, China

**DOI:** 10.3389/fcimb.2021.653983

**Published:** 2021-08-13

**Authors:** Qian Zeng, Shuzhen Xiao, Feifei Gu, Weiping He, Qing Xie, Fangyou Yu, Lizhong Han

**Affiliations:** ^1^Department of Laboratory Medicine, Ruijin Hospital, Shanghai Jiao Tong University School of Medicine, Shanghai, China; ^2^Department of Clinical Microbiology, Ruijin Hospital, Shanghai Jiao Tong University School of Medicine, Shanghai, China; ^3^Department of Infectious Diseases, Translational Laboratory of Liver Diseases, Ruijin Hospital, Shanghai Jiao Tong University School of Medicine, Shanghai, China; ^4^Department of Clinical Laboratory Medicine, Shanghai Pulmonary Hospital, Tongji University School of Medicine, Shanghai, China

**Keywords:** female, urinary tract infection, uropathogenic *Escherichia coli*, antimicrobial resistance, molecular epidemiology

## Abstract

Urinary tract infection (UTI) is one of the most common bacterial infections and UTI is the most common extraintestinal infectious disease entity in women worldwide. Uropathogenic *Escherichia coli* (UPEC) is the leading cause of UTI. While antimicrobial resistance has emerged as one of the principal problems of UTI, little is known about the epidemiology of UPEC isolated from female patients in Shanghai. This study aimed to describe the antimicrobial resistance and molecular epidemiology of UPEC isolated from female patients in Shanghai, China. UPEC isolates were collected from female patients from July 2019 to June 2020 in Shanghai and a total of 151 isolates were obtained randomly. Antimicrobial susceptibility testing was performed using the disk diffusion method. Multilocus sequencing type, phylogenetic groups, antimicrobial resistance genes, and virulence genes were detected by polymerase chain reaction. In our study, no carbapenem-resistant isolates were found, but fluoroquinolone-resistant and multi-drug resistant UPEC accounted for 62.25% and 42.38%, respectively. The phylogenetic group B2 (58.94%) predominated, followed by phylogenetic group D (26.49%). The most prevalent sequence type was ST1193 (25.83%), which was first reported in Shanghai. The rate of extended-spectrum β-lactamase (ESBL)-positive isolates was 39.74% and the dominant ESBL genotype was *bla*_CTX-M-14_ (21/60), followed by *bla*_CTX-M-55_ (12/60). Mutations in *gyrA* were detected in the majority of fluoroquinolone-resistant isolates (90/94), followed by *parC* (85/94) and *parE* (71/94). The *aac (3) -IIa* was also found in 85% of aminoglycoside resistance isolates. Among 151 UPEC isolates, the common virulence genes were *csgA* (97.35%), *fimH* (92.72%), *sitA* (82.12%), and *malX* (65.56%). In conclusion, the high antimicrobial resistance of UPEC isolated from female patients, harboring a series of virulence genes, are troublesome for medical practitioners in Shanghai. At present, the prevalent ST1193 and emerging *bla*_CTX-M-55_ make UTI therapy more challenging.

## Introduction

Urinary tract infection (UTI) is one of the most common bacterial infections. Almost 150 million UTIs occur per year worldwide, resulting in more than 6 billion dollars in direct health care expenditure (Stamm and Norrby, 2001). According to CHINET surveillance results of bacterial resistance in China, the pathogens isolated from urine ranked only second to those isolated from the respiratory tract (http://www.chinets.com/). Due to anatomical differences, UTI is the most common extraintestinal infectious disease entity in women worldwide, nearly one-third of women will develop a UTI requiring antibiotic treatment by age 24, and more than one-half of women will experience at least once UTI by the end of life (Dielubanza and Schaeffer, 2011). Uropathogenic *Escherichia coli* (UPEC) is the leading cause of UTI, accounting for 70-95% of community-acquired UTI and 50% of nosocomial UTI (Xia et al., 2017).

Antibiotic therapy is the most critical treatment for UTI, however, high resistance to cephalosporins and fluoroquinolones has become a major concern in recent years (Jean et al., 2016). Extended-spectrum β-lactamases (ESBLs) is one of the primary mechanisms conferring resistance to β-lactam antibiotics (Sheu et al., 2018). Quinolone resistance is associated closely with mutations in the quinolone resistance-determining regions (QRDRs) of DNA gyrase and topoisomerase IV, and plasmid-mediated quinolone resistance (PMQR) genes (Cao et al., 2011). Resistance to aminoglycosides may occur due to methylation of 16S rRNA and aminoglycoside modifying enzymes(AME) and the most common mechanism of resistance to aminoglycosides are AMEs. (Ramirez and Tolmasky, 2010). Additionally, UPEC have evolved to carry a range of virulence genes that promote colonizing and survival in the urethra, such as fimbriae with adhesin tips, protections, production of toxins, leading to recurrent UTI (Kucheria et al., 2005).

Based on previous studies, the antibiotic resistance of UTI-relevant gram-negative bacteria in China is relatively high (Zhang et al., 2019). But the distribution of antimicrobial susceptibility and molecular epidemiology vary greatly across regions, and little is known about the epidemiology of UPEC isolated from female patients in Shanghai. Therefore, in this study, we reported multilocus sequencing type (MLST), phylogenetic group, antimicrobial susceptibility, and the prevalence of antimicrobial resistance and virulence genes of UPEC isolated from female patients in Shanghai.

## Materials and Methods

### Setting and Strain Collection

Our study was conducted at Shanghai Ruijin Hospital, a general tertiary hospital with about 2100 beds, serving a population of approximately 24 million in a large metropolitan region. The Nephrology Department of Ruijin Hospital has a high reputation in China, ranking first in various medical indicators. The number of outpatient and emergency treatments in this department is about 190,000 per year.

In our study, a total of 604 isolates from episodes of UTI in 604 female patients were collected between July 2019 and June 2020 at Shanghai Ruijin Hospital. All isolates were identified by matrix-assisted laser desorption ionization-time of flight mass spectrometer (bioMérieux, Marcy-l’Étoile, France) and stored at -80°C in broth containing 30% glycerol until used. According to the age (age ≥60 years or age <60 years) of patients and clinical departments visited by patients, they were divided into four layers: elderly outpatients (*n* =184), non-elderly outpatients (*n* =160), elderly inpatients (*n* =160) and non-elderly inpatients (*n* =100). Stratified sampling was used to extract 25% isolates from each layer and a total of 151 UPEC isolates were obtained by random sampling in Microsoft Office Excel 2010 (Microsoft Corporation, Redmond, WA, USA).

### Antimicrobial Susceptibility Test and Confirmatory Test for ESBL

Susceptibility testing used the disk diffusion method and susceptibility profiles were interpreted according to the 2020 CLSI criteria, except interpretation for tigecycline was based on the criteria of the European Committee on Antimicrobial Susceptibility Testing (EUCAST) (CLSI, 2020). The following antimicrobial agents were tested: ceftazidime (CAZ), cefotaxime (CTX), cefazolin (KZ), meropenem (MEM), imipenem (IMP), ciprofloxacin (CIP), levofloxacin (LEV), gentamicin (GEN), amikacin (AK), tobramycin (TOB), doxycycline (DOX), minocycline (MI), tigecycline (TIG), aztreonam (ATM), fosfomycin (FOS), nitrofurantoin (AHD), trimethoprim/sulfamethoxazole (SXT), piperacillin/tazobactam (TZP), ceftazidime/avibactam (CAZ/AVI). A double-disk synergy test (cefotaxime and ceftazidime disks with and without clavulanic acid) was used as a confirmatory test for ESBL producers. *E.coli* ATCC25922, *E.coli* ATCC35218, *Klebsiella pneumoniae* ATCC700603, and *Pseudomonas aeruginosa* ATCC27853 were used for quality control.

### Multilocus Sequencing Type

Seven conserved housekeeping genes (*adk, fumC,gyrB, icd, mdh, purA, and recA*) were utilized to determine MLST and protocols are available at https://enterobase.readthedocs.io/en/latest/mlst/mlst-legacy-info-ecoli.html. Aligning the sequences and estimating the phylogenetic tree were conducted in MEGAX (Hall, 2013). For the purpose of defining clades throughout this work, clades were defined using the bootstrap method with at least 1,000 bootstrap replication. The Interactive Tree of Life website was used to generate an image of a phylogenetic tree including metadata (https://itol.embl.de).

### Phylogenetic Group Analysis

Phylogenetic analysis of *E.coli* is composed of four main phylogenetic groups (A, B1, B2, and D), and this study used a simple and rapid phylogenetic grouping technique based on triplex PCR with a combination of two genes (*chuA and yjaA*) and an anonymous DNA fragment (*TspE4C2*) described previously (Clermont et al., 2000).

### Identification of Antimicrobial Resistance Genes and Mutations

Two fresh colonies were resuspended in 1mL of distilled water and lysed at 100°C for 15 min, then centrifuged at 14 000 rpm for 10 min. The supernatant was used as a source of template DNA for amplification. Sixty isolates were positive in the confirmatory test for ESBLs producers and polymerase chain reaction (PCR) was detected for *bla*_TEM_, *bla*_SHV_, *bla*_CTX-M (-1, -9, group)_, *bla*_OXA(-1,-2,-10 group)_, *bla*_VEB_, *bla*_PER_ (Wang et al., 2016). Ninety-four UPEC isolates were resistant to fluoroquinolones and *qnrA, qnrB, qnrC, qnrD, qnrS, qepA, oqxAB, aac (6’) Ib-cr, gyrA, gyrB, papC, papE* were detected (Chen et al., 2012; Pitondo-Silva et al., 2015). Forty strains of *E.coli* were not susceptible to aminoglycoside and PCR used for detecting *aac (3) -IIa, armA, rmtB* (Fernández-Martínez et al., 2015; Ayad et al., 2016). The sequences of primers for PCR amplification were presented in **Table S1**. All PCR fragments were sequenced and the gene types were identified by comparing them to sequences in GenBank (https://blast.ncbi.nlm.nih.gov/Blast.cgi). Mutations in *gyrA*, *gyrB*, *parC*, and *parE* were compared with the sequence of the reference genes *E. coli* K-12 (GenBank NC_000913.3).

### Detection of Virulence Genes

All 151 UPEC were screened for the presence of common virulence genes (Rodriguez-Siek et al., 2005), including type-1 fimbriae gene (*fimH)*, afimbrial adhesins gene (*afa*), pyelonephritis-associated pili gene (*papA*, *papC*), temperature-sensitive hemagglutinin gene (*tsh*), invasion of brain endothelium gene (*ibeA*), curli fimbriae gene (*csgA*), transport gene of the haemolysin operon (*hlyD*), putative iron transport gene (*sitA*), increased serum survival gene (*iss*), and pathogenicity island marker gene (*malX*) (Naziri et al., 2020).

### Statistical Analysis

SPSS Statistics 26 system (IBM, Armonk, NY) was used for statistical analysis. Continuous variables are presented as mean ± SD or median with interquartile range. Categorical variables were compared by Chi-square test, Fisher’s exact test, or Continuity correction in different situations. A two-tailed *p* values less than 0.05 were considered statistically significant.

## Results

### Patient Demographics and Genetic Relationship of UPEC

**Figure 1:**
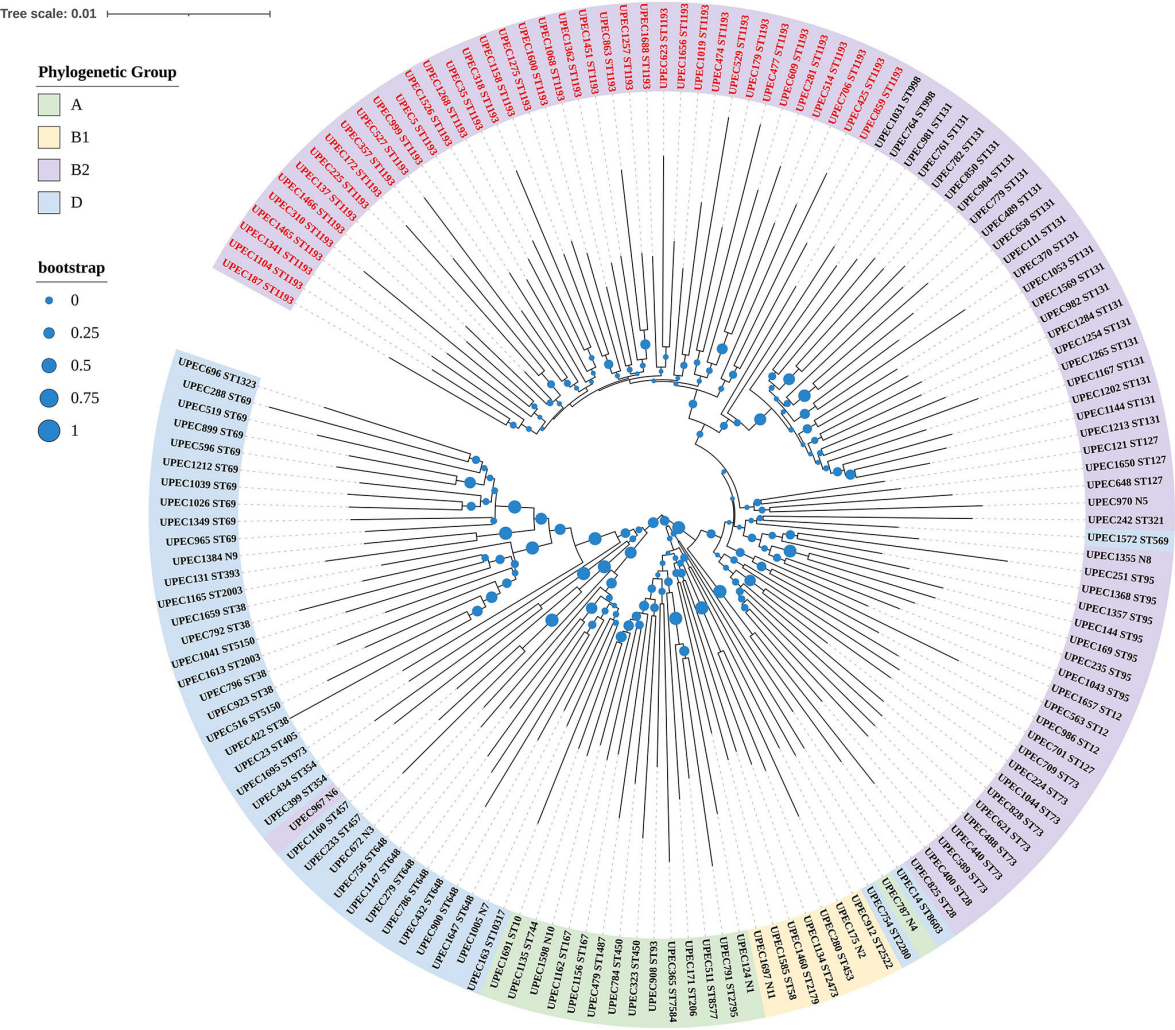
Phylogenetic tree of UPEC isolated from female patients based on seven conserved housekeeping genes. In the tree, the UPEC of different colored regions belonged to different phylogenetic groups, and the numbers after UPEC were made up when we collected the isolates clinically. There were 50 different sequence types and N1 to N11 were represented for new ST types. The most prevalent sequence type was ST1193 and UPEC identified as ST1193 were marked in bright red.

In this study, the age of 151 female patients ranged from 19 to 94y, the median age was 62y and the quartile range was 19y. As for the genetic relationship, phylogenetic group B2 (58.94%) predominated, followed by phylogenetic group D (26.49%), phylogenetic group A (9.93%), and phylogenetic group B1 (4.64%). We also identified 50 different sequence types including 11 new sequence types (**Figure 1**). The most prevalent sequence type was ST1193 (*n*=39), followed by ST131(*n*=20), all those isolates also belonged to phylogenetic group B2. In addition, we found non-ESBL isolates were more common in phylogenetic group B2, however, ESBL and multi-drug resistance (MDR, nonsusceptibility to ≥1 agent in ≥3 antimicrobial categories) (Magiorakos et al., 2012) isolates were more frequent in the phylogenetic group D.

### Antimicrobial Resistance Profiles

All 151 UPEC isolates were susceptible to CAZ/AVI, MEM, IMP, TIG and more than 95% of the isolates were susceptible to TZP, AK, FOS, AHD, but the resistant rates to CTX, KZ, CIP, LEV, and SXT were 39.07%, 41.06%, 62.25%, 55.63%, and 42.38%, respectively. We found 60 UPEC isolates were positive in the confirmatory test for ESBL producers, 94 isolates were fluoroquinolone-resistant (FQ-R) and 64 isolates were MDR. In this study, ESBL isolates exhibited statistically lower susceptible rates to CAZ, CTX, KZ, CIP, LEV, TOB, MI, ATM than non-ESBL isolates (*p*<0.05). As well, the resistant rates of CAZ, CTX, KZ, CIP, LEV, GEN, TOB and ATM in FQ-R isolates were higher than fluoroquinolone-susceptible (FQ-S) isolates (*p*<0.05). Besides, the isolates belonging to phylogenetic group B2 had significantly higher susceptible rates to CAZ, CTX, KZ, MI, and ATM than isolates of phylogenetic group D (*p*<0.05). Surprisingly, all ST1193 isolates were resistant to ciprofloxacin and levofloxacin, which was quite different from non-ST1193 isolates (*p*<0.05). The antibiotic susceptible rates are shown in **Table 1**. In addition, there was no statistically significant difference in antibiotic sensitivity among elderly outpatients, non-elderly outpatients, elderly inpatients, and non-elderly inpatients (*p>*0.05) and the raw data were presented in **Table S2**.

**Table 1:** Antibiotic susceptible rates of 151 UPEC isolates.

Antibiotics	TOTAL(n = 151)	ESBL(n = 60)	non-ESBL(n = 91)	FQ-R(n = 94)	FQ-S(n = 53)	ST1193(n = 39)	non-ST1193(n = 112)	B2(n = 89)	D (n = 40)
Ceftazidime	82.12%	55.00%	100.00%	76.60%	90.57%	87.18%	80.36%	88.76%	70.00%
Cefotaxime	60.26%	0.00%	100.00%	48.94%	79.25%	66.67%	58.04%	69.66%	37.50%
Cefazolin	58.94%	0.00%	97.80%	48.94%	75.47%	66.67%	56.25%	69.66%	35.00%
Piperacillin/tazobactam	96.69%	93.33%	98.90%	95.74%	98.11%	97.44%	96.43%	95.51%	97.50%
Ceftazidime/avibactam	100.00%	100.00%	100.00%	100.00%	100.00%	100.00%	100.00%	100.00%	100.00%
Ciprofloxacin	36.42%	18.33%	48.35%	0.00%	100.00%	0.00%	49.11%	35.96%	37.50%
Levofloxacin	37.75%	21.67%	48.35%	3.19%	100.00%	0.00%	50.89%	33.71%	42.50%
Gentamicin	76.82%	71.67%	80.22%	69.15%	90.57%	71.79%	78.57%	79.78%	75.00%
Amikacin	98.01%	95.00%	100.00%	96.81%	100.00%	100.00%	97.32%	100.00%	95.00%
Tobramycin	78.81%	70.00%	84.62%	73.40%	90.57%	74.36%	80.36%	78.65%	80.00%
Meropenem	100.00%	100.00%	100.00%	100.00%	100.00%	100.00%	100.00%	100.00%	100.00%
Imipenem	100.00%	100.00%	100.00%	100.00%	100.00%	100.00%	100.00%	100.00%	100.00%
Doxycycline	68.21%	61.67%	72.53%	65.96%	69.81%	79.49%	64.29%	76.40%	62.50%
Minocycline	91.39%	85.00%	95.60%	88.30%	96.23%	94.87%	90.18%	96.63%	82.50%
Tigecycline	100.00%	100.00%	100.00%	100.00%	100.00%	100.00%	100.00%	100.00%	100.00%
Aztreonam	72.19%	30.00%	100.00%	63.83%	86.79%	79.49%	69.64%	78.65%	60.00%
Fosfomycin	98.01%	96.67%	98.90%	96.81%	100.00%	97.44%	98.21%	97.75%	100.00%
Nitrofurantoin	99.34%	98.33%	100.00%	98.94%	100.00%	100.00%	99.11%	100.00%	100.00%
Trimethoprim/sulfamethoxazole	56.95%	48.33%	62.64%	50.00%	66.04%	53.85%	58.04%	62.92%	52.50%

Comparison of two rates was conducted in different pairwise comparisons (ESBL vs non-ESBL, FQ-R vs FQ-S, ST1193 vs non-ST1193, phylogenetic group B2 vs phylogenetic group D) and shaded areas indicate a significant difference (p < 0.05).

### Characterization of Resistance Genes and Mutations

Of the phenotypic ESBL producing strains, 57 out of 60 (95%) harbored at least one of the *bla* genes, containing *bla*_TEM_, *bla*_SHV_, *bla*_CTX-M (-1, -9, group)_, *bla*_OXA(-1,-2,-10 group)_, *bla*_VEB_, *bla*_PER_. Among ESBL genes, *bla*_CTX-M-14_ accounted for 35%, followed by *bla*_CTX-M-55_ (20%), *bla*_CTX-M-27_ (18.33%) and *bla*_CTX-M-15_ (13.33%). The gene *bla*_TEM_ or *bla*_OXA-1_ were detected along with gene *bla*_CTX-M_ in 16 ESBL producing isolates and gene *bla*_TEM_ was detected in 13 isolates, *bla*_OXA-1_ was detected in 3 isolates. Interestingly, the gene *bla*_CTX-M-64_ was only detected in 3 isolates of ST1193, *bla*_CTX-M-69_ was detected in one ST95 isolate and rare *bla*_CTX-M-123_ was detected in one ST69 isolate. We also found that there is no statistical difference in the distribution of β-lactamase genes between FQ-R and FQ-S isolates or between phylogenetic group B2 and group D isolates.

As for fluoroquinolone resistance, PMQR was detected in a relatively small number of FQ-R isolates (*n* = 11, 11.70%), and 4 isolates harbored *acc (6 ‘) Ib-cr* also carried *bla*_CTX-M_ and *aac (3) -IIa* gene. Mutations in gyrA gene (*n* = 90, 95.74%) of DNA gyrase, and mutations in *parC* gene (*n* = 85, 90.43%) and *parE* gene (*n* =71, 75.53%) of topoisomerase IV were found in majority of FQ-R isolates. In this study, the *gyrA* mutation merely occurred at positions Ser83 and Asp87, the mutations in *parC* occurred at position Ser80 in all cases and mutations of *parC* also occurred at positions Ser57, Glu84, Lys113 in a small number of isolates, and 95.77% of mutations in *parE* occurred at positions Leu416, Ser458 or Ile529. We found that the distribution of the fluquinolone resistant gene was not statistically significant between ESBL and non-ESBL isolates, except the mutation of *parE* at Leu416Phe occurred more frequently in non-ESBL isolates (*p*<0.05). Comparing to non-ST1193 isolates, the ST1193 isolates harbored the same four nonsynonymous mutations in *gyrA* (D87N and S83L), *parC* (S80I), and *parE* (L416F) (*p*<0.05). The mutation rate of *parE* was higher and mutation of *parE* at Leu416 was more frequent in the phylogenetic group B2 (*p*<0.05), but mutation of *parE* at Ser458 was more common in phylogenetic group D (*p*<0.001).

In aminoglycoside resistance isolates, *aac (3) -IIa* was found in 85% of strains. But in the methylation of 16S rRNA, *rmtB* was only found in one UPEC isolate and this isolate was resistant to AK, GEN, and TOB. The distribution of resistance genes and mutations are shown in **Table 2**.

**Table 2:** Prevalence of different resistance genes and their combinations with antibiotic resistance profiles in UPEC isolated from female patients.

Phylogenetic group	ST	*bla* gene	QNR	AME/16S rRNA methylases	Antibiotic resistance profiles	Number(*n* = 151)
A	10	/	/	/	SXT-DOX	1
	93	/	*gyrA*: S83L, D87N, *parC*: S80I	*aac(3’)-IIa*	SXT-CIP-LEV-GEN	1
	167	*bla*_CTX-M-55_	*qnrS-1, qepA, gyrA*: S83L, D87N, *parC*: S80I, *parE*: S458A	*aac(3’)-IIa, rmtB*	ATM-SXT-CAZ-CTX-KZ-CIP-LEV-GEN-AK-TOB-DOX-MI	1
		/	/	*aac(3’)-IIa*	GEN-dox	1
	206	*bla*_CTX-M-55_	*gyrA*: S83L, D87N, *parC*: S80I	/	ATM-SXT-CAZ-CTX-KZ-CIP-LEV-DOX-MI	1
	450	*bla*_CTX-M-15_, *bla*_OXA-1_	*aac(6’)-Ib-cr, gyrA*: S83L, D87N, *parC*: S80I, *parE*: S458A	*aac(3’)-IIa*	ATM-SXT-CAZ-CTX-KZ-CIP-LEV-GEN-TOB-dox	1
		/	*gyrA*: S83L, D87N, *parC*: S80I	/	CIP-LEV	1
	744	/	*qnrS-1*, *gyrA*: S83L, D87N, *parC*: S80I	/	SXT-CIP-LEV-DOX-MI	1
	1487	*bla*_CTX-M-14_	*gyrA*: S83L, D87N, *parC*: S80I	/	ATM-ahd-CTX-KZ-CIP-LEV-DOX	1
	2795	/	/	/	KZ	1
	7584	*bla*_TEM-150_, *bla*_CTX-M-27_	*gyrA*: S83L, D87N, *parC*: S80I	*aac(3’)-IIa*	ATM-SXT-CTX-KZ-CIP-LEV-GEN-TOB-dox	1
	8577	*bla*_CTX-M-15_	*qnrS-1*	/	ATM-caz-CTX-KZ-CIP	1
	N1	/	/	/	/	1
	N10	/	/	/	SXT-dox	1
	N4	/	*qnrS-1*	/	CIP-lev	1
B1	58	/	/	/	SXT-dox	1
	453	/	*gyrA*: S83L, D87N, *parC*: S80I	*aac(3’)-IIa*	SXT-CIP-LEV-GEN-TOB-DOX	1
	2179	*bla*_TEM-1_, *bla*_CTX-M-55_	*gyrA*: S83L, *parC*: S80I	/	ATM-caz-CTX-KZ-CIP	1
	2473	/	/	/	/	1
	2522	/	*qnrS-1*	/	SXT-CIP-lev-DOX	1
	N11	*bla*_CTX-M-14_	*gyrA*: S83L, D87N, *parC*: S80I, *parE*: L416F	/	FOS-SXT-CTX-KZ-CIP-LEV-gen-TOB	1
	N2	/	/	/	SXT	1
B2	12	*bla*_CTX-M-14_	/	/	CTX-KZ	1
		/	/	/	SXT	1
		/	/	/	/	1
	28	/	/	/	dox	1
		/	/	/	SXT-DOX	1
	73	/	*gyrA*: S83L	/	CIP-lev	1
		/	/	/	DOX-mi-tzp	1
		/	/	/	/	6
	95	*bla*_CTX-M-69_	/	/	ATM-CAZ-CTX-KZ	1
		/	/	/	SXT	1
		/	/	/	SXT-dox	1
		/	/	/	dox	1
		/	/	/	/	3
	127	/	*gyrA*: S83L	/	CIP-lev	1
		/	/	/	/	3
	131	*bla*_TEM-1_, *bla*_CTX-M-55_	*gyrA*: S83L, *parE*: S458A, I529L	*aac(3’)-IIa*	ATM-CAZ-CTX-KZ-CIP-LEV-GEN-tob	1
		*bla*_TEM-1_, *bla*_CTX-M-14_	*gyrA*: S83L, D87N, *parC*: S80I, *parE*: L445H	/	ATM-CTX-KZ-CIP-LEV-tzp	1
		*bla*_TEM-1_, *bla*_CTX-M-27_	*gyrA*: S83L, *parE*: I529L	*aac(3’)-IIa*	ATM-SXT-caz-CTX-KZ-CIP-lev-GEN-tob-DOX	1
		*bla*_CTX-M-15_, *bla*_OXA-1_	*aac(6’)-Ib-cr, gyrA*: S83L,D87N, *parC*: S80I, E84V, *parE*: I529L	*aac(3’)-IIa*	ATM-SXT-caz-CTX-KZ-CIP-LEV-GEN-TOB	1
		*bla*_CTX-M-15_, *bla*_OXA-1_	*aac(6’)-Ib-cr, gyrA*: S83L,D87N, *parC*: S80I, E84V, *parE*: I529L	*aac(3’)-IIa*	SXT-caz-CTX-KZ-CIP-LEV-GEN-TOB-tzp	1
		*bla*_CTX-M-15_	/	/	atm-SXT-CTX-KZ	1
		*bla*_CTX-M-14_	*gyrA*: S83L, D87N, *parC*: S80I, E84V, *parE*: I529L	*aac(3’)-IIa*	atm-SXT-CTX-KZ-CIP-LEV-GEN-TOB	1
		*bla*_CTX-M-14_	*gyrA*: S83L, D87N, *parC*: S80I, E84V, *parE*: I529L	/	CTX-KZ-CIP-LEV	1
		*bla*_CTX-M-14_	*gyrA*: S83L, D87N, *parC*: S80I, E84V, *parE*: I529L	/	atm-SXT-CTX-KZ-CIP-LEV-DOX	1
		*bla*_CTX-M-27_	*gyrA*: S83L, D87N, *parC*: S80I, E84V, *parE*: I529L	/	ATM-SXT-CTX-KZ-CIP-LEV-DOX	1
		*bla*_CTX-M-27_	/	/	ATM-CTX-KZ-cip-lev-TOB	1
		/	*gyrA*: S83L, D87N, *parC*: S80I, E84V, *parE*: I529L	/	CIP-LEV	2
		/	*gyrA*: S83L, D87N, *parC*: S80I, E84V, *parE*: I529L	/	SXT-CIP-LEV-DOX	1
		/	*gyrA*: S83L, D87Y, *parC*: S80I, E84V, *parE*: S458A	/	SXT-CIP-LEV	1
		/	/	*aac(3’)-IIa*	GEN-lev-tob	1
		/	/	*aac(3’)-IIa*	SXT-GEN-tob-DOX	1
		/	/	/	SXT-DOX	1
		/	/	/	lev	1
		/	/	/	/	1
	321	/	/	/	/	1
	998	/	*gyrA*: S83L	/	CIP-lev-DOX	1
		/	/	/	/	1
	1193	*bla*_TEM-1_, *bla*_CTX-M-55_	*gyrA*: S83L, D87N, *parC*: S80I, *parE*: L416F	/	ATM-SXT-CAZ-CTX-KZ-CIP-LEV-DOX	1
		*bla*_TEM-1_, *bla*_CTX-M-64_	*gyrA*: S83L, D87N, *parC*: S80I, *parE*: L416F	*aac(3’)-IIa*	ATM-SXT-CAZ-CTX-KZ-CIP-LEV-GEN-TOB	1
		*bla*_CTX-M-15_	*qnrS-1*, *gyrA*: S83L, D87N, *parC*: S80I, *parE*: L416F	/	CTX-KZ-CIP-LEV	1
		*bla*_CTX-M-55_	*gyrA*: S83L, D87N, *parC*: S80I, *parE*: L416F	/	ATM-CAZ-CTX-KZ-CIP-LEV	1
		*bla*_CTX-M-55_	*gyrA*: S83L, D87N, *parC*: S80I, *parE*: L416F	/	ATM-CTX-KZ-CIP-LEV	1
		*bla*_CTX-M-64_	*gyrA*: S83L, D87N, *parC*: S80I, *parE*: L416F	*aac(3’)-IIa*	ATM-SXT-CAZ-CTX-KZ-CIP-LEV-GEN-TOB	1
		*bla*_CTX-M-64_	*gyrA*: S83L, D87N, *parC*: S80I, *parE*: L416F	/	ATM-CAZ-CTX-KZ-CIP-LEV-tzp	1
		*bla*_CTX-M-14_	*gyrA*: S83L, D87N, *parC*: S80I, *parE*: L416F	/	atm-CTX-KZ-CIP-LEV	1
		*bla*_CTX-M-27_	*gyrA*: S83L, D87N, *parC*: S80I, *parE*: L416F	/	atm-CTX-KZ-CIP-LEV	1
		*bla*_CTX-M-27_	*gyrA*: S83L, D87N, *parC*: S80I, *parE*: L416F	/	FOS-CTX-KZ-CIP-LEV	1
		*bla*_CTX-M-27_	*gyrA*: S83L, D87N, *parC*: S80I, *parE*: L416F	/	SXT-CTX-KZ-CIP-LEV-dox	1
		*bla*_CTX-M-27_	*gyrA*: S83L, D87N, *parC*: S80I, *parE*: L416F	/	SXT-CTX-KZ-CIP-LEV-DOX-mi	1
		/	*gyrA*: S83L, D87N, *parC*: S80I, *parE*: L416F	*aac(3’)-IIa*	CIP-LEV-GEN	1
		/	*gyrA*: S83L, D87N, *parC*: S80I, *parE*: L416F	*aac(3’)-IIa*	SXT-CIP-LEV-GEN-tob	3
		/	*gyrA*: S83L, D87N, *parC*: S80I, *parE*: L416F	*aac(3’)-IIa*	SXT-CIP-LEV-GEN-TOB	3
		/	*gyrA*: S83L, D87N, *parC*: S80I, *parE*: L416F	*aac(3’)-IIa*	SXT-CIP-LEV-GEN-TOB-dox	1
		/	*gyrA*: S83L, D87N, *parC*: S80I, *parE*: L416F	/	CIP-LEV	12
		/	*gyrA*: S83L, D87N, *parC*: S80I, *parE*: L416F	/	sxt-CIP-LEV-DOX-MI	1
		/	*gyrA*: S83L, D87N, *parC*: S80I, *parE*: L416F	/	SXT-CIP-LEV	2
		/	*gyrA*: S83L, D87N, *parC*: S80I, *parE*: L416F	/	SXT-CIP-LEV-DOX	2
		/	*gyrA*: S83L, D87N, *parC*: S80I, *parE*: L416F	/	SXT-CTX-KZ-CIP-LEV-DOX	1
		/	*gyrA*: S83L, D87N, *parC*: S80I, *parE*: L416F	*aac(3’)-IIa*	CIP-LEV-GEN-TOB	1
	N5	*bla*_CTX-M-14_	/	/	atm-CTX-KZ-TOB	1
	N6	/	*gyrA*: D87Y, *parC*: S80I, *parE*: S458A	/	FOS-CIP-LEV	1
	N8	/	/	/	/	1
D	38	*bla*_TEM-1_, *bla*_CTX-M-14_	/	*aac(3’)-IIa*	SXT-CTX-KZ-GEN-tob-DOX	1
		*bla*_TEM-150_, *bla*_CTX-M-14_	/	*aac(3’)-IIa*	SXT-CTX-KZ-GEN-tob	1
		*bla*_CTX-M-15_	/	/	ATM-caz-CTX-KZ	1
		*bla*_CTX-M-14_	*aac(6’)-Ib-cr*, *gyrA*: S83L,D87N, *parC*: S80I	*aac(3’)-IIa*	ATM-SXT-caz-CTX-KZ-CIP-LEV-GEN-ak-TOB-DOX-MI-tzp	1
		*bla*_CTX-M-14_	*gyrA*: S83L, D87G, *parC*: S80I	*aac(3’)-IIa*	SXT-CTX-KZ-CIP-lev-GEN	1
	69	*bla*_CTX-M-123_	/	/	ATM-CAZ-CTX-KZ	1
		*bla*_CTX-M-55_	/	/	atm-CTX-KZ	1
		*bla*_CTX-M-27_	*gyrA*: S83L, D87N, *parC*: S80I	/	atm-SXT-CTX-KZ-CIP-LEV-DOX-MI	1
		/	*gyrA*: S83L, *parC*: S80I	/	CIP	1
		/	/	*aac(3’)-IIa*	SXT-KZ-GEN-DOX-mi	1
		/	/	/	SXT-TOB-DOX	1
		/	/	/	SXT-caz-ctx-KZ	1
		/	/	/	/	2
	354	*bla*_TEM-150_, *bla*_CTX-M-14_	*gyrA*: S83L, D87N, *parC*: S80I, E84G	/	ATM-SXT-CTX-KZ-CIP-LEV-DOX	1
		*bla*_CTX-M-14_	*gyrA*: S83L, D87N, *parC*: S80I, E84G	/	ATM-SXT-CAZ-CTX-KZ-CIP-LEV-DOX-mi	1
	393	/	*gyrA*: S83L, D87N, *parC*: S80I, *parE*: L416F	*aac(3’)-IIa*	SXT-CIP-LEV-GEN-TOB-dox	1
	405	/	*gyrA*: S83L, D87N, *parC*: S80I, K113R, *parE*: S458A	*aac(3’)-IIa*	CIP-LEV-GEN-TOB	1
	457	*bla*_TEM-150_, *bla*_CTX-M-14_	*gyrA*: S83L, D87N, *parC*: S80I, *parE*: S458A	*aac(3’)-IIa*	SXT-CTX-KZ-CIP-LEV-GEN-TOB	1
		*bla*_CTX-M-55_	*gyrA*: S83L, D87Y, *parC*: S80I, *parE*: S458A	/	ATM-CAZ-CTX-KZ-CIP-LEV	1
	569	/	/	/	/	1
	648	*bla*_CTX-M-15_	*gyrA*: S83L, D87N, *parC*: S80I, *parE*: S458A	/	ATM-SXT-CAZ-CTX-KZ-CIP-LEV-AK-DOX-mi	1
		*bla*_CTX-M-14_	*gyrA*: S83L, D87N, *parC*: S80I, *parE*: S458A	/	CTX-KZ-CIP-LEV	2
		/	*qepA*, *gyrA*: S83L, D87N, *parC*: S80I, *parE*: S458A	*aac(3’)-IIa*	SXT-CIP-LEV-GEN-DOX	1
		/	*gyrA*: S83L, D87N, *parC*: S80I, *parE*: S458A	/	ATM-CAZ-CTX-KZ-CIP-LEV	1
		/	*gyrA*: S83L, D87N, *parC*: S80I, *parE*: S458A	/	SXT-CIP-LEV	1
		/	/	/	SXT	1
	973	*bla*_CTX-M-55_	/	/	ATM-SXT-caz-CTX-KZ-DOX	1
	1323	/	/	/	/	1
	2003	*bla*_CTX-M-55_	*gyrA*: S83L, D87N, *parC*: S80I	/	ATM-SXT-caz-CTX-KZ-CIP-LEV-DOX-MI	1
		*bla*_CTX-M-14_	*gyrA*: S83L, D87N, *parC*: S80I, *parE*: L502F	/	CTX-KZ-CIP-LEV	1
	2280	*bla*_CTX-M-14_	/	/	ATM-CTX-KZ-CIP-LEV	1
	5150	*bla*_TEM-1_, *bla*_CTX-M-55_	*gyrA*: S83L, D87N, *parC*: S80I	*aac(3’)-IIa*	ATM-caz-CTX-KZ-CIP-LEV-GEN	1
		/	/	/	cip	1
	8603	/	/	/	/	1
	10317	/	*gyrA*: S83L, D87N, *parC*: S80I, *parE*: S458A	/	CIP-LEV	1
	N3	*bla*_TEM-1_, *bla*_CTX-M-27_	*gyrA*: S83L, D87N, *parC*: S80I, *parE*: S458A	/	atm-CTX-KZ-CIP-LEV-TOB-DOX	1
	N7	*bla*_CTX-M-14_	*gyrA*: S83L, D87N, *parC*: S80I, *parE*: S458A	/	CTX-KZ-CIP-LEV-DOX-MI	1
	N9	*bla*_CTX-M-27_	*gyrA*: S83L, D87N, *parC*: S57T, S80I, *parE*: L416F	/	atm-SXT-caz-CTX-KZ-CIP-LEV-DOX	1

N1 to N11 were represented for new ST types. In the antibiotic resistance profile, the uppercase of antibiotics are presented the UPEC isolate is resistant to this antibiotic, and the lowercase of antibiotics are presented the UPEC isolate is intermediate resistant to this antibiotic.

### Identification of Virulence Genes

In this study, the highest proportion of virulence genes were adhesion genes. We found that *csgA* was detected in nearly all UPEC isolates (97.35%), followed by well-known *fimH* (92.72%), *sitA* (82.12%), and *malX* (65.56%). The proportion of the same virulence genes was similar between ESBL and non-ESBL isolates, except *hlyD* was detected more in non-ESBL isolates (*p*=0.013). However, compared to FQ-R isolates, FQ-S isolates harbored more virulence genes, as *papA*, *papC*, *tsh*, *hlyD*, *ibeA*, *iss* (*p*<0.05). Phylogenetic group B2 isolates were detected more in *papC*, *hlyD*, *sitA*, *malX* than phylogenetic group D isolates (*p*<0.05). Based on the results of the comparison, it is worth thinking about a reverse correlation between the virulence factors and antimicrobial resistance of UPEC. We also found that the percentage of *fimH*, *csgA*, *sitA*, *malX* in ST1193 isolates is even over 90%, but few ST1193 isolate detected *papA*, *papC*, *afa*, *tsh*, *hlyD*, *iss*, which was different from non-ST1193 isolates. The distribution of virulence genes is shown in **Table 3**.

**Table 3:** Distribution of virulence genes among UPEC isolated from female patients.

Phylogenetic group	ST	Virulence factors	Number(*n* = 151)
A	10	*fimH, afa, csgA*	1
	93	*fimH, csgA, sitA, iss*	1
	167	*fimH, csgA*	1
		*papA, papC, csgA, hlyD*	1
	206	*fimH, csgA*	1
	450	*fimH, papA, papC, csgA*	1
		*fimH, csgA*	1
	744	*fimH, tsh, csgA, sitA, iss*	1
	1487	*fimH, csgA*	1
	2795	*fimH, csgA*	1
	7584	*fimH, csgA, sitA, iss*	1
	8577	*fimH, csgA*	1
	N1	*fimH, csgA*	1
	N10	*fimH, csgA, sitA*	1
	N4	*csgA*	1
B1	58	*fimH, csgA, sitA, iss*	1
	453	*fimH, csgA, sitA, iss*	1
	2179	*fimH, csgA, sitA, iss*	1
	2473	*fimH, csgA*	1
	2522	*fimH, csgA, sitA, iss*	1
	N11	*fimH, csgA*	1
	N2	*fimH, csgA, sitA, iss*	1
B2	12	*fimH, papC, csgA, hlyD, sitA, malX*	3
	28	*fimH, ibeA, csgA, sitA, malX*	1
		*fimH, ibeA, csgA, malX*	1
	73	*fimH, papA, papC, csgA, hlyD, sitA, malX*	6
		*fimH, csgA, hlyD, sitA, malX*	2
	95	*fimH, papA, papC, tsh, csgA, sitA, iss, malX*	3
		*fimH, papA, papC, ibeA, csgA, hlyD, sitA, iss, malX*	1
		*fimH, papA, papC, csgA, hlyD, sitA, iss, malX*	1
		*fimH, papA, papC, csgA, sitA, iss, malX*	1
		*fimH, papC, tsh, csgA, sitA, iss, malX*	1
	127	*fimH, papA, papC, csgA, hlyD, sitA, malX*	2
		*fimH, papC, csgA, hlyD, sitA, malX*	1
		*fimH, papC, hlyD*	1
	131	*fimH, afa, csgA, sitA, malX*	4
		*fimH, papA, papC, csgA, hlyD, sitA, malX*	3
		*fimH, papA, papC, csgA, sitA, malX*	1
		*fimH, papA, papC, hlyD, sitA*	1
		*fimH, papC, csgA, hlyD, sitA, malX*	1
		*fimH, csgA, sitA, malX*	7
		*fimH, csgA, malX*	2
		*fimH, sitA, malX*	1
	321	*fimH, tsh, ibeA, csgA, sitA, malX*	1
	998	*fimH, papA, papC, ibeA, csgA, hlyD, sitA, iss, malX*	2
	1193	*fimH, ibeA, csgA, sitA, malX*	1
		*fimH, csgA, sitA, malX*	33
		*fimH, csgA, sitA*	2
		*csgA, sitA, malX*	3
	N5	*fimH, csgA, sitA, malX*	1
	N6	*fimH, papA, papC, csgA, hlyD, sitA, malX*	1
	N8	*fimH, ibeA, csgA, malX*	1
D	38	*fimH, afa, csgA, sitA*	1
		*fimH, csgA, sitA*	3
		*fimH, csgA*	1
	69	*fimH, papA, papC, csgA, sitA*	1
		*fimH, tsh, csgA, sitA, iss*	2
		*fimH, csgA, sitA*	6
	354	*fimH, ibeA, csgA*	1
		*fimH, csgA, sitA, malX*	1
	393	*fimH, papA, papC, csgA, sitA*	1
	405	*fimH, csgA, sitA*	1
	457	*fimH, csgA, sitA, iss, malX*	1
		*fimH, csgA, sitA, malX*	1
	569	*fimH, ibeA, csgA, sitA*	1
	648	*fimH, papA, papC, sitA, malX*	1
		*fimH, tsh, csgA, sitA, iss*	1
		*fimH, csgA, sitA, malX*	1
		*csgA, sitA, malX*	2
		*csgA, malX*	2
	973	*fimH, csgA, sitA, iss*	1
	1323	*fimH, csgA, hlyD, sitA, malX*	1
	2003	*fimH, afa, csgA, sitA*	1
		*fimH, afa, csgA*	1
	2280	*fimH, csgA, malX*	1
	5150	*fimH, afa, csgA, sitA*	1
		*fimH, csgA, sitA*	1
	8603	*fimH, csgA*	1
	10317	*fimH, csgA, malX*	1
	N3	*afa, csgA, sitA, malX*	1
	N7	*csgA, malX*	1
	N9	*fimH, papA, papC, csgA, sitA*	1

N1 to N11 were represented for new ST types.

## Discussion

This study has reported on the antimicrobial resistance and molecular epidemiology of UPEC isolated from female patients in Shanghai. Nearly 78% of female patients ranged in age from 42y to 81y and there were slightly more outpatients than inpatients. The resistance rates of UPEC strains to a majority of commonly used antimicrobials in our study were high and a global spread of MDR bacterial strains seems an inevitable reality with increasing individual mobility (Frost et al., 2019). Considering resistance rates of UPEC in fluoroquinolones, cephalosporins, and trimethoprim-sulfamethoxazole were so high, that these antibiotics should be used more carefully (Gupta et al., 2017; Sun et al., 2020).

In recent years and throughout most of the world, CTX-M type genes replaced SHV and TEM as the most common ESBLs gene, particularly in ESBL-producing *E.coli* (Rodríguez-Baño et al., 2006). Previous surveys have described that CTX-M-14 was the predominant ESBL genotype in China (Yu et al., 2007), and a high prevalence of CTX-M-15 was reported initially in China fifty years ago (Liu et al., 2009). However, a recent study revealed that CTX-M-55 had spread rapidly (Xia et al., 2014; Zhang et al., 2014). In our study, we reported that CTX-M-14 was predominant among CTX-M genes, followed by CTX-M-55. The high prevalence of CTX-M-55 detected in *E.coli* has not previously been reported in Shanghai.

PMQR contains three different mechanisms, including five major groups of *Qnr* determinants (*QnrA, QnrB, QnrC, QnrD, and QnrS*), main transferable efflux pumps (*QepA and OqxAB*), and antibiotic modification mediated by *AAC (6 ‘) Ib-Cr* (Ruiz, 2019). QRDRs consist of both mutations in *gyrA* and *gyrB* of DNA gyrase and mutations in *parC* and *parE* genes of topoisomerase IV (Liu et al., 2012). The plasmid-mediated mechanisms provide low-level resistance but facilitate the selection of higher-level resistance and make infection by pathogens containing PMQR harder to treat (Jacoby et al., 2014). For isolates, double mutations in *gyrA* were a precondition for conferring a resistant phenotype, and additional mutations of *parC* or *parE* confer high levels of fluoroquinolones (Moon et al., 2010). Besides, mutations at different sites of *parE* confer different levels of FQ resistance, the Leu416Phe substitution in *parE* occurred in isolates with lower levels of FQ resistance than Ser458Ala mutation or Ile529Leu alteration in *par*E (Azargun et al., 2019). Therefore, we can explain the results of the total detection rate in FQ-resistance and quinolone-resistant (Qnr) genes between different pairwise comparisons. We also revealed that FQ resistance within ST1193 was of chromosomal origin (S80I in *parC*, L416F in *parE*, and D87N and S83L in *gyrA*). In our study, *aac (3) -IIa* was detected in 85% of UPEC isolates resistant to aminoglycoside, and *rmtB* gene was detected in only one isolate, which confer high level resistant in aminoglycosides (Bartoloni et al., 2016).

The pathogenesis of UPEC involves multiple virulence factors including toxins, adhesins, secretion, and iron acquisition systems to resist urinary flow, trigger host bacterial cell signaling pathways, and establish infection (Alteri and Mobley, 2012). According to our findings, the *csgA* gene was the most frequent virulence-associated gene and the high prevalence of *csgA*, *fimH*, *sitA*, *malX* in UPEC has also been reported in other literature (Ejrnæs et al., 2011; Ochoa et al., 2016; Paniagua-Contreras et al., 2018). Our findings support the hypothesis that antibiotic-susceptible isolates mostly belong to the phylogenetic group B2 and were associated with higher virulence factor prevalence than antibiotic-resistant isolates, which were typically associated with group D (Er et al., 2015; Zhao et al., 2015). We also found that nearly all ST1193 isolates carried *fimH*, *csgA*, *sitA*, and *malX*, but few ST1193 isolates detected pyelonephritis associated genes and other virulence genes (*afa*, *tsh*, *hlyD*, *iss*). It was been reported that the prevalent signature F-type plasmid was observed within ST1193 among globally extraintestinal pathogenic *E.coli* and this plasmid had been discovered conferring enhanced bladder colonization and invasion (Johnson et al., 2019). Based on this information, we suspect that ST1193 has its own signature F-type plasmid mediated in either clonal virulence or fitness and this phenomenon warrants further study.

Over the past two decades, the *E.coli* sequence type 131 (ST131) clone has emerged as an important human pathogen worldwide and has been recognized as a pandemic clone (López-Cerero et al., 2014). In addition, the *E.coli* ST131 clone appears to be a consistent predictor of treatment failure in UTI (Can et al., 2015). However, since 2012, reports from individual hospitals in China, Norway, America, South Korea, and Australia have documented an epidemic of quinolone-resistant *E.coli* ST1193 (Tchesnokova et al., 2019). The fluoroquinolone-resistant ST1193 of *E. coli*, from the ST14 clonal complex (STc14) within phylogenetic group B2, has appeared recently. Therefore, we have to deal with ST1193 isolates more cautiously due to the alarming detection rate of ST1193. In our study, all ST1193 isolates were resistant to ciprofloxacin and levofloxacin, and the proportion of ST1193 is the highest, exceeding ST131. Although ST1193 isolates were from female patients in this study, the rate of UPEC isolated from female patients was 5.4 times that of male patients between July 2019 and June 2020 in Ruijing hospital, and our results were also representative to a certain extent. To our knowledge, this is the first article that reported a high prevalence of *E. coli* ST1193 in Shanghai.

In terms of sequence types and the predominant ESBL types of UPEC, the results of this study are similar to the results of a recent study conducted in Zhejiang Province, China (Quan et al., 2021). These results reveal the spread of ST1193 and CTX-M-55 of UPEC in China. In the past decade, considerable studies have suggested that the worldwide increase of *E. coli* producing CTX-M-15 enzymes was associated with an epidemic clone ST131 (Peirano and Pitout, 2010). However, we did not have enough data to support the relationship between transition in sequence type (such as the spread of ST1193) and transition in prevalent ESBL types (such as the increasing CTX-M-55) of UPEC, which is the main limitation of our study. More evidence is needed to investigate the reasons for the epidemiological changes of UPEC.

In conclusion, considering high resistance to the most widely used antibacterial agents (i.e. fluoroquinolones, cephalosporins, and trimethoprim-sulfamethoxazole) for treatment of UTI caused by UPEC isolates, we suggest that clinicians should consider the susceptibility results of the UPEC isolated from female patients when choosing antibiotics, and we recommend nitrofurantoin and fosfomycin as empirical antibiotics. At present, the high antimicrobial resistance of UPEC isolated from female patients, carrying a series of virulence genes are troublesome, and present problems for medical practitioners in Shanghai. The prevalent ST1193 and emerging *bla*_CTX-M-55_ make UTI therapy more challenging. Therefore, we must continually explore the latest changes in the epidemiology of UPEC isolates to assist clinical treatment.

## Data Availability Statement

The original contributions presented in the study are included in the article/**Supplementary Material**. Further inquiries can be directed to the corresponding authors.

## Ethics Statement

This study was approved by the Ethics Committee of Ruijin Hospital affiliated with Shanghai Jiao Tong University School of Medicine. Because this retrospective study only experimented on bacteria and did not affect the patients adversely, the Review Board exempted the study from requesting informed consent.

## Author Contributions

LH and FY conceived and designed the experiments. QZ performed the experiments. QZ analyzed the data. SX, QX, FG, and WH contributed reagents/materials/analysis tools. QZ wrote the manuscript. LH and SX edited the manuscript. All authors contributed to the article and approved the submitted version.

## Funding

This study was financially supported by the Shanghai Municipal Key Clinical Specialty (shslczdzk01103).

## Conflict of Interest

The authors declare that the research was conducted in the absence of any commercial or financial relationships that could be construed as a potential conflict of interest.

## Publisher’s Note

All claims expressed in this article are solely those of the authors and do not necessarily represent those of their affiliated organizations, or those of the publisher, the editors and the reviewers. Any product that may be evaluated in this article, or claim that may be made by its manufacturer, is not guaranteed or endorsed by the publisher.
