# Supplementary material for: Antimicrobial Resistance and Molecular Epidemiology of Uropathogenic Escherichia coli Isolated From Female Patients in Shanghai, China
Source: Front Cell Infect Microbiol. 2021 Aug 13;11:653983. doi: 10.3389/fcimb.2021.653983 (PMC8414883; doi:10.3389/fcimb.2021.653983)
Supplement: Supplementary file 1 [file Table_1.doc]

**Table S1** Sequences of primers for PCR amplification.

| **Primer** | **Sequences (5'-3')** | **Product (bp)** |
| --- | --- | --- |
| chuA-F | GACGAACCAACGGTCAGGAT | 279 |
| chuA-R | TGCCGCCAGTACCAAAGACA |
| yjaA-F | TGAAGTGTCAGGAGACGCTG | 211 |
| yjaA-R | ATGGAGAATGCGTTCCTCAAC |
| TspE4.C2-F | GAGTAATGTCGGGGCATTCA | 152 |
| TspE4.C2-R | CGCGCCAACAAAGTATTACG |
| SHV-F | TGGTTATGCGTTATATTCGCC | 865 |
| SHV-R | GGTTAGCGTTGCCAGTGCT |
| TEM-F | ATAAAATTCTTGAAGACGAAA | 1080 |
| TEM-R | GACAGTTACCAATGCTTAATC |
| CTX-1-F | CGTCACGCTGTTGTTAGGAA | 780 |
| CTX-1-R | ACGGCTTTCTGCCTTAGGTT |
| CTX-9-F | TATTGGGAGTTTGAGATGGT | 932 |
| CTX-9-R | TCCTTCAACTCAGCAAAAGT |
| OXA1-F | CTGTTGTTTGGGTTTCGCAAG | 440 |
| OXA1-R | CTTGGCTTTTATGCTTGATG |
| OXA2-F | CAGGCGCYGTTCGYGATGAGTT | 233 |
| OXA2-R | GCCYTCTATCCAGTAATCGCC |
| OXA10-F | GTCTTTCRAGTACGGCATTA | 822 |
| OXA10-R | GATTTTCTTAGCGGCAACTTA |
| VEB-F | GCGGTAATTTAACCAGA | 961 |
| VEB-R | GCCTATGAGCCAGTGTTC |
| PER-F | AGTCAGCGGCTTAGATA | 978 |
| PER-R | CGTATGAAAAGGACAATC |
| qnrA-F | AGAGGATTTCTCACGCCAGG | 619 |
| qnrA-R | GCAGCACTATKACTCCCAAGG |
| qnrB-F | GGMATHGAAATTCGCCACTG | 264 |
| qnrB-R | TTTGCYGYYCGCCAGTCGAA |
| qnrC-F | GGGTTGTACATTTATTGAATC | 447 |
| qnrC-R | TCCACTTTACGAGGTTCT |
| qnrD-F | CGAGATCAATTTACGGGGAATA | 582 |
| qnrD-R | AACAAGCTGAAGCGCCTG |
| qnrS-F | GCAAGTTCATTGAACAGGCT | 428 |
| qnrS-R | TCTAAACCGTCGAGTTCGGCG |
| qepA-F | CTGCAGGTACTGCGTCATG | 403 |
| qepA-R | CGTGTTGCTGGAGTTCTTC |
| **Primer** | **Sequences (5'-3')** | **Product (bp)** |
| oqxA-F | GACAGCGTCGCACAGAATG | 339 |
| oqxA-R | GGAGACGAGGTTGGTATGGA |
| oqxB-F | CGAAGAAAGACCTCCCTACCC | 240 |
| oqxB-R | CGCCGCCAATGAGATACA |
| aac (6') -Ib-cr-F | TTGCGATGCTCTATGAGTGGCTA | 482 |
| aac (6') -Ib-cr-R | CTCGAATGCCTGGCGTGTTT |
| gyrA-F | CATGAACGTATTGGGCAATG | 305 |
| gyrA-R | CCGTACCGTCATAGTTATCC |
| gyrB-F | CTCCTCCCAGACCAAAGACA | 447 |
| gyrB-R | TCACGACCGATACCACAGCC |
| parC-F | AAACCTGTTCAGCGCCGCATT | 395 |
| parC-R | GTGGTGCCGTTAAGCAAA |
| parE-F | GCCCAGCGCCGTATGCGTGC | 621 |
| parE-R | GTTCGGATCAAGCGTGGTTT |
| aac (3') -IIa-F | GGCAATAACGGAGGCGCTTCAAAA | 563 |
| aac (3') -IIa-R | TTCCAGGCATCGGCATCTCATACG |
| armA-F | CAAATGGATAAGAATGATGTT | 777 |
| armA-R | TTATTTCTGAAATCCACT |
| rmtB-F | TCAACGATGCCCTCACCTC | 459 |
| rmtB-R | GCAGGGCAAAGGTAAAATCC |
| fimH-F | TCGAGAACGGATAAGCCGTGG | 508 |
| fimH-R | GCAGTCACCTGCCCTCCGGTA |
| papA-F | ATGGCAGTGGTGTTTTGGTG | 717 |
| papA-R | CGTCCCACCATACGTGCTCTTC |
| malX-F | GGACATCCTGTTACAGCGCGCA | 925 |
| malX-R | TCGCCACCAATCACAGAAGAAC |
| papC-F | GACGGCTGTACTGCAGGGTGTGGCG | 328 |
| papC-R | ATATCCTTTCTGCAGGGATGCAATA |
| afa-F | GCTGGGCAGCAAACTGATAACTCTC | 750 |
| afa-R | CATCAAGCTGTTTGTTCGTCCGCCG |
| csgA-F | ACTCTGACTTGACTATTACC | 200 |
| csgA-R | AGATGCAGTCTGGTCAAC |
| tsh-F | GGGAAATGACCTGAATGCTGG | 420 |
| tsh-R | CCGCTCATCAGTCAGTACCAC |
| hlyD-F | CTCCGGTACGTGAAAAGGAC | 904 |
| hlyD-R | GCCCTGATTACTGAAGCCTG |
| ibeA-F | AGGCAGGTGTGCGCCGCGTAC | 171 |
| ibeA-R | TGGTGCTCCGGCAAACCATGC |
| **Primer** | **Sequences (5'-3')** | **Product (bp)** |
| sitA-F | AGGGGGCACAACTGATTCTCG | 608 |
| sitA-R | TACCGGGCCGTTTTCTGTGC |
| iss-F | GTGGCGAAAACTAGTAAAACAGC | 760 |
| iss-R | CGCCTCGGGGTGGATAA |

| **Antibiotics** | **elderly outpatients (n=46)** | **non-elderly outpatients**  **(n=40)** | **elderly inpatients**  **(n=40)** | **non-elderly inpatients**  **(n=25)** |
| --- | --- | --- | --- | --- |
| Ceftazidime | 80.43% | 80.00% | 87.50% | 80.00% |
| Cefotaxime | 58.70% | 60.00% | 65.00% | 56.00% |
| Cefazolin | 58.70% | 57.50% | 65.00% | 52.00% |
|  |  |  |  |  |
| Piperacillin/tazobactam | 97.83% | 95.00% | 95.00% | 100.00% |
| Ceftazidime/avibactam | 100.00% | 100.00% | 100.00% | 100.00% |
|  |  |  |  |  |
| Ciprofloxacin | 30.43% | 40.00% | 40.00% | 36.00% |
| Levofloxacin | 30.43% | 42.50% | 37.50% | 44.00% |
|  |  |  |  |  |
| Gentamicin | 76.09% | 77.50% | 75.00% | 80.00% |
| Amikacin | 100.00% | 97.50% | 95.00% | 100.00% |
| Tobramycin | 78.26% | 75.00% | 75.00% | 92.00% |
|  |  |  |  |  |
| Meropenem | 100.00% | 100.00% | 100.00% | 100.00% |
| Imipenem | 100.00% | 100.00% | 100.00% | 100.00% |
|  |  |  |  |  |
| Doxycycline | 78.26% | 55.00% | 67.50% | 72.00% |
| Minocycline | 97.83% | 82.50% | 92.50% | 92.00% |
| Tigecycline | 100.00% | 100.00% | 100.00% | 100.00% |
|  |  |  |  |  |
| Aztreonam | 71.74% | 75.00% | 72.50% | 68.00% |
|  |  |  |  |  |
| Fosfomycin | 97.83% | 97.50% | 97.50% | 100.00% |
|  |  |  |  |  |
| Nitrofurantoin | 100.00% | 100.00% | 97.50% | 100.00% |
|  |  |  |  |  |
| Trimethoprim/sulfamethoxazole | 63.04% | 52.50% | 52.50% | 60.00% |

TABLE S2 Antibiotic susceptible rates of four types of patients.

Comparison of antibiotic susceptible rates were conducted among elderly outpatients, non-elderly outpatients, elderly inpatients and non-elderly inpatients, and the differences among them were not statistically significant (*p*>0.05), and only the susceptible rate in doxycycline and minocycline of non-elderly outpatients are lower than elderly outpatients in pairwise comparison(*p*<0.05).
